# New-onset and relapse of nephrotic syndrome following COVID-19 vaccination: a questionnaire survey in Japan

**DOI:** 10.1007/s10157-022-02231-y

**Published:** 2022-05-15

**Authors:** Naoki Nakagawa, Shoichi Maruyama, Naoki Kashihara, Ichiei Narita, Yoshitaka Isaka

**Affiliations:** 1 Joint Research Team from Japanese Society of Nephrology and the Progressive Renal Diseases Research, Research on Intractable Disease, from the Ministry of Health, Labour and Welfare of Japan, Special Study Group for Nephrotic Syndrome, Tokyo, Japan; 2 Division of Cardiology, Nephrology, Pulmonology and Neurology, Department of Internal Medicine, Asahikawa Medical University, 2-1-1-1 Midorigaoka-higashi, Asahikawa, Japan; 3 Department of Nephrology, Nagoya University Graduate School of Medicine, Nagoya, Japan; 4 Department of Nephrology and Hypertension, Kawasaki Medical School, Kurashiki, Japan; 5 Division of Clinical Nephrology and Rheumatology, Kidney Research Center, Niigata University Graduate School of Medical and Dental Sciences, Niigata, Japan; 6 Department of Nephrology, Osaka University Graduate School of Medicine, Suita, Japan

**Keywords:** SARS-CoV-2 vaccination, Coronavirus 2019, Nephrotic syndrome, Minimal change disease, mRNA vaccination

## Abstract

**Background:**

Recent clinical reports indicate a correlation between new-onset and relapse of nephrotic syndrome (NS) following coronavirus 2019 (COVID-19) vaccination in patients with glomerular diseases. However, there are no reports of a nationwide survey on NS following COVID-19 vaccination in Japan.

**Methods:**

We conducted a web-based survey of council members of the Japanese Society of Nephrology (581 members, 382 facilities) to elucidate the relationship between COVID-19 vaccination and new-onset and relapse of NS.

**Results:**

Following COVID-19 vaccination, 27 patients (male: 15, 55.6%) with new-onset (*n* = 6) and relapse (*n* = 21) of NS were reported. Of them, 12 (44.4%) patients were diagnosed with minimal change disease at the occurrence of NS. Five patients developed a slight increase in serum creatinine levels; however, none progressed to severe renal dysfunction.

**Conclusion:**

Our findings clarify the clinical features of new-onset and relapse of NS following COVID-19 vaccination. Although there was no obvious progression to severe renal dysfunction, clinicians and pathologists should be aware that NS is a potential adverse effect of the vaccines.

## Introduction

Since its emergence at the end of 2019, the coronavirus disease (COVID-19) has spread globally and resulted in major crises in healthcare systems and the global economy [1, 2]. Several vaccines have been developed to control the spread of COVID-19 and reduce the severity of the disease and the risk of death [3–5]. In Japan, healthcare workers were initially vaccinated with an mRNA vaccine (BNT162b2 [COMIRNATY], Pfizer-BioNTech; Pfizer, New York, NY, USA and BioNTech, Mainz, Germany) [3] on February 17, 2021. To date, a total of approximately two hundred and forty million vaccines have been administrated in Japan [6].

Non-serious local and systemic reactions are common among adolescents and adults who receive a COVID-19 vaccine, while reports of severe adverse events like myocarditis are rare [7]. Recently, several studies have reported the incidence of new-onset and relapse of nephrotic syndrome (NS) following COVID-19 vaccination in patients with glomerular diseases, especially those with minimal change disease (MCD) [8–14]. Furthermore, several studies have reported the incidence of gross hematuria following COVID-19 vaccination in patients with immunoglobulin A nephropathy (IgAN) [15–17] and IgA vasculitis [18]. Most recently, a clinical survey of gross hematuria associated with COVID-19 vaccination using a web-based questionnaire in Japan reported the clinical features of gross hematuria in 27 patients following COVID-19 vaccination [19]. Therefore, investigating the frequency and clinical features of new-onset and relapse of NS following COVID-19 vaccination in Japan is vital for the clinical management of NS during the current pandemic. To this end, a joint research team from the Japanese Society of Nephrology and the Progressive Renal Diseases Research, Research on Intractable Disease, from the Ministry of Health, Labour and Welfare of Japan, conducted a clinical survey of new-onset and relapse of NS associated with COVID-19 vaccination using a web-based questionnaire.

## Methods

The first survey comprised a web-based questionnaire emailed to the council members of the Japanese Society of Nephrology (581 members in 382 facilities) between August 31 and September 30, 2021. The questionnaire inquired about the cases of new-onset and relapse of NS that were observed following COVID-19 vaccination and their outcomes (Table 1). Subsequently, between march 3 and march 10, 2022, a second survey was emailed to the members who reported cases of elevated serum creatinine levels after new-onset and relapse of NS. The second survey enquired about the course of elevated serum creatinine levels and pathological diagnosis if a renal biopsy was performed. All data were analyzed using IBM SPSS v.26.0 (SPSS, Chicago, IL, USA).

**Table 1:** Contents of the questionnaire in the first survey

Question number	Questions	Response
Q1-1	How about this episode?	1. New-onset
		2. Relapse
Q1-2	How old is this patient (years)?	1. ≤ 19
		2. 20–29
		3. 30–39
		4. 40–49
		5. 50–59
		6. 60–69
		7. ≥ 70
Q1-3	What is the patient's sex?	1. Male
		2. Female
Q1-4	Has this patient undergone a renal biopsy? If yes, what was their diagnosis?	1. Diagnosed by renal biopsy
		2. Did not perform a renal biopsy
		3. Others (If performed renal biopsy, please describe the details in this section.)
Q1-5	Check all the treatments used in this patient before this episode	1. No treatment
		2. Oral corticosteroids
		3. Steroid pulse therapy
		4. Immunosuppressive therapy (except for rituximab)
		5. Rituximab
		6. RAS-I
		7. Antiplatelet drugs
		8. Others
Q2-1	What type of vaccine was used in this patient?	1. COMIRNATY intramuscular injection (Pfizer-BioNTech)
		2. COVID-19 vaccine moderna intramuscular injection (Moderna/Takeda)
		3. VAXZEVRIA intramuscular injection (AstraZeneca)
		4. Others
Q2-2	After what vaccination did you point out the proteinuria?	1. After first-dose vaccination
		2. After second-dose vaccination
		3. Both first-dose and second-dose vaccination
		4. Others
Q2-3	How many days after vaccination did the proteinuria appear?	1. ≤ 1 day
		2. 2–3 days
		3. 4–7 days (almost 1 week)
		4. 8–14 days (almost 2 weeks)
		5. 15–28 days (almost 3–4 weeks)
		6. Others
Q2-4	Did you start/increase the dose of steroids or increase the dose of immunosuppressive drugs after the appearance of proteinuria?	1. Started the dose of steroids
		2. Increased the dose of steroids
		3. Increase the dose of immunosuppressive drugs
		4. Only follow-up
		5. Others (If you have increased the dose of immunosuppressive drugs, please describe the details in the "Other" section.)
Q2-5	How long did the proteinuria continue?	1. ≤ 1 day
		2. 2–3 days
		3. 4–7 days (almost 1 week)
		4. Over 8 days
		5. Others
Q2-6	Did an adverse reaction to the vaccination occur in the patient with the proteinuria?	1. Did not experience an adverse reaction
		2. Unknown
		3. Fever (≥ 37.5 ℃)
		4. Headache
		5. General fatigue
		6. Chills
		7. Muscle pain
		8. Joint pain
		9. Others
Q3-1	Did this patient have proteinuria of qualitative (+) or higher or 0.3 g/day (g/g Cr) or higher prior to the vaccination?	1. Yes
		2. No
Q3-2	How about the amount of urinary protein at the time of this episode?	1. 1.0 g/day (g/g Cr) ≤ proteinuria < 3.5 g/day (g/g Cr)
		2. ≥ 3.5 g/day (g/g Cr)
Q3-3	Was there a worsening of renal function after this episode?	1. Yes
		2. No
		3. Others
Q3-4	Did this patient have hematuria of urinary occult blood qualitative (+) or higher or urinary red blood cell 5/HPF or higher prior to the vaccination?	1. Yes
		2. No
Q3-5	Was there any appearance or exacerbation of hematuria in this episode?	1. Yes
		2. No
		3. Others

*COVID-19* coronavirus disease 2019, *IgA* immunoglobulin A, *HPF* high-power field, *RAS-I* renin–angiotensin system inhibitor

## Results

**Fig. 1:**
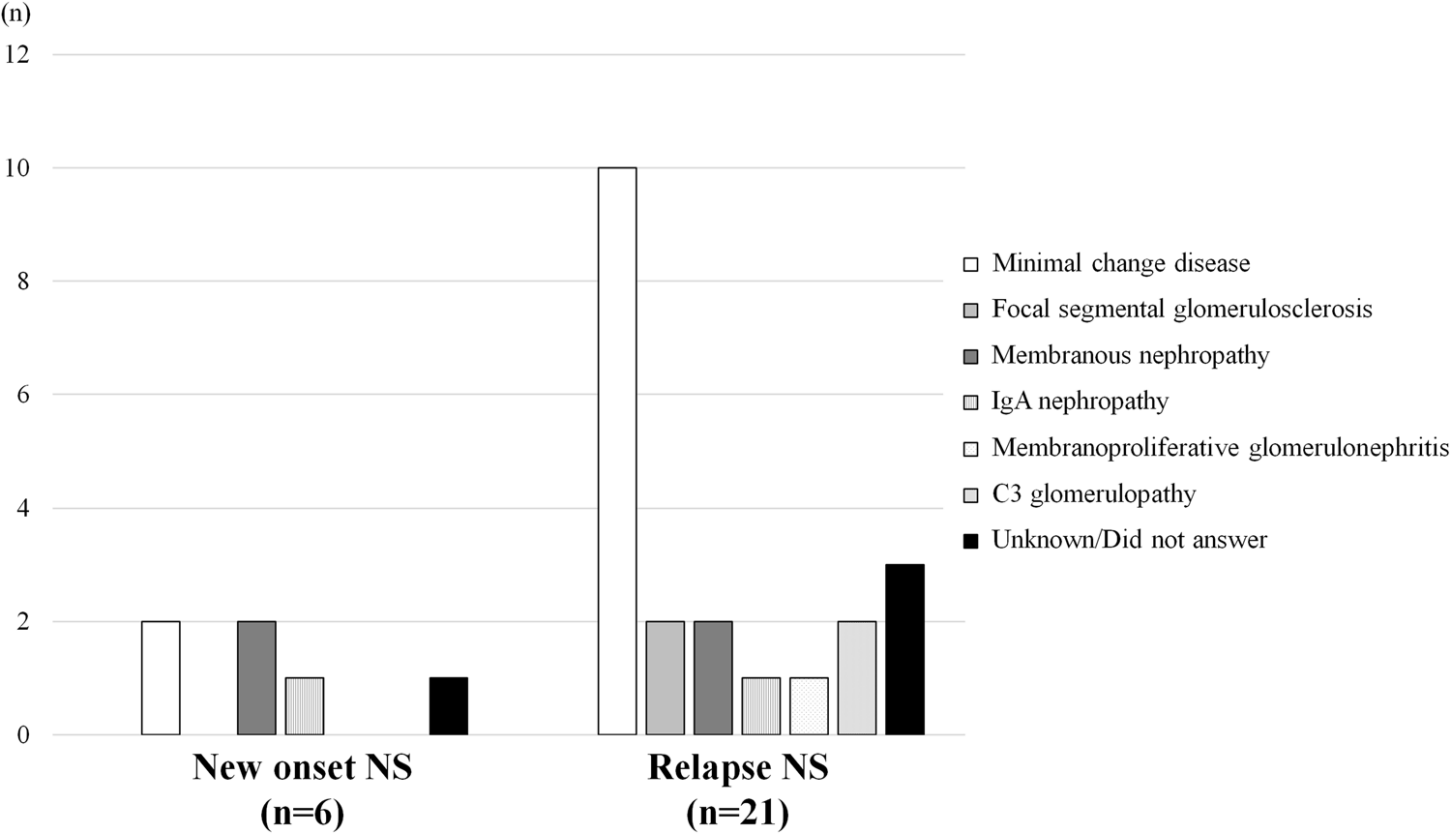
Disease categories of new-onset and relapse of nephrotic syndrome following coronavirus (COVID)-19 vaccination

In the first survey, 55 members (response rate: 14.4% of facilities) reported 27 cases of new-onset (*n* = 6) and relapse (*n* = 21) of NS following COVID-19 vaccination. The baseline characteristics of the patients with new-onset and relapse of NS are summarized in Table 2. Most patients were aged over 70 years (25.9%), and 48.1% were over 60 years of age. Male patients comprised 55.6% of the study population. Furthermore, 23 (85.2%) cases were observed following vaccination with BNT162b2 (Pfizer-BioNTech) with an onset of NS after the first (*n* = 8) and second (*n* = 15) doses of the vaccine. Of the 27 patients, 12 (44.4%) were diagnosed with MCD; 2 (7.4%), with focal segmental glomerulosclerosis; 2 (7.4%), with IgAN, 4 (14.8%), with membranous nephropathy; 1 (3.7%), with membranoproliferative glomerulonephritis; and 2 (7.4%), with C3 glomerulopathy (Table 2; Fig. 1).

**Table 2:** Baseline characteristics of patients with new-onset and relapse of nephrotic syndrome after receiving COVID-19 vaccination

Characteristic	All cases (*n* = 27)	New-onset (*n* = 6)	Relapse (*n* = 21)
Age (years)			
≤ 19	3	0	3
20–29	4	0	4
30–39	3	0	3
40–49	2	1	1
50–59	2	0	2
60–69	6	1	5
≥ 70	7	4	3
Sex			
Female	12	2	10
Male	15	4	11
Treatments before this event (multiple answers allowed)			
No treatment	10	6	4
Oral corticosteroid	15	0	15
Steroid pulse therapy	3	0	3
Immunosuppressive therapy (except for rituximab)	10	0	10
Rituximab	3	0	3
RAS-I	7	0	7
Antiplatelet drugs	1	0	1
Type of the vaccine			
COMIRNATY Intramuscular Injection (Pfizer-BioNTech)	23	5	18
COVID-19 Vaccine Moderna Intramuscular Injection (Moderna/Takeda)	1	0	1
Unknown	3	1	2
Vaccination dose			
First dose	8	1	7
Second dose	18	4	14
Both first and second doses	1	1	1
Histopathological diagnoses revealed by the kidney biopsy			
Minimal change disease	12	2	10
Focal segmental glomerulosclerosis	2	0	2
Membranous nephropathy	4	2	2
IgA nephropathy	2	1	1
Membranoproliferative glomerulonephritis	1	0	1
C3 glomerulopathy	2	0	2
Unknown/did not answer	4	1	3
Adverse reactions (multiple answers allowed)			
Fever (≥ 37.5 ℃)	5	0	5
Fatigue	8	0	8
Headache	1	0	1
Chills	0	0	0
Muscle pain	6	2	4
Pain at the application site	1	0	1
Joint pain	2	0	2
None	2	0	2
Unknown	8	4	4

*COVID-19* coronavirus disease 2019, *RAS-I* renin–angiotensin system inhibitor

**Fig. 2:**
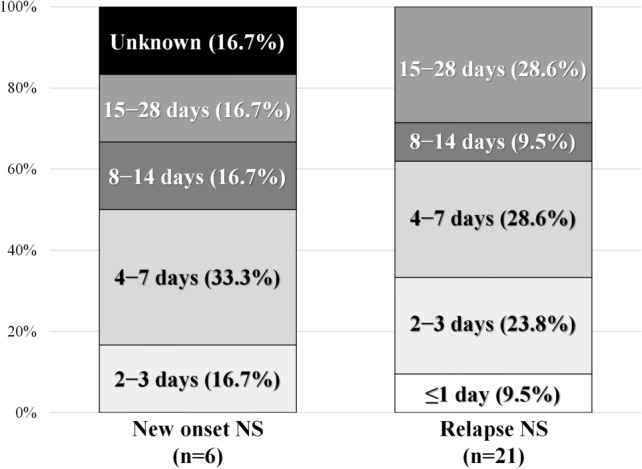
Duration between coronavirus (COVID)-19 vaccination and the incidence of new-onset and relapse of nephrotic syndrome

**Fig. 3:**
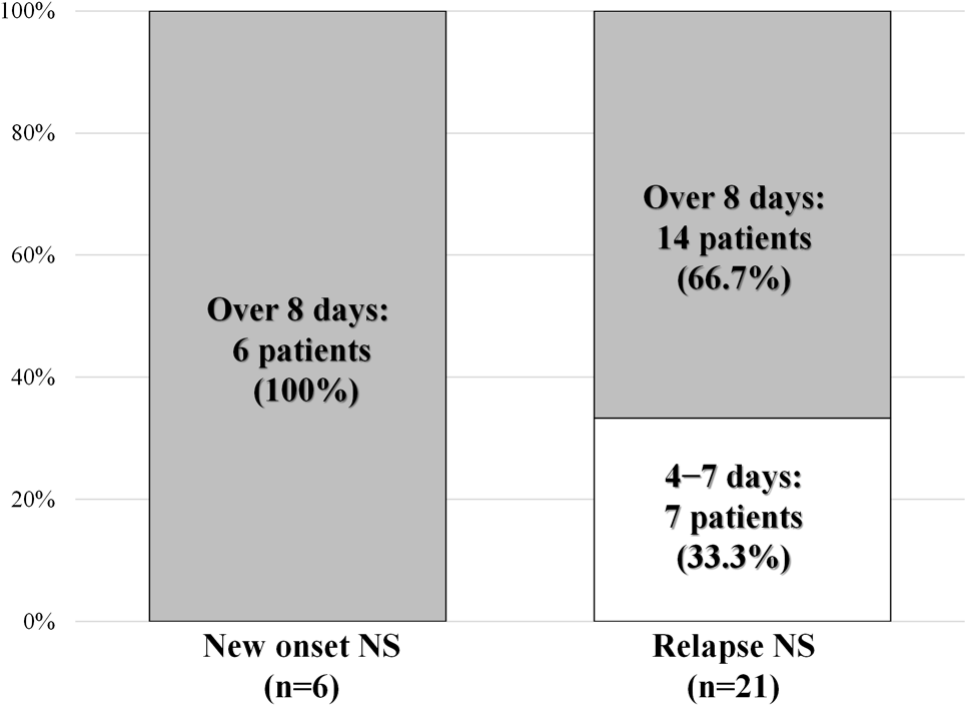
Duration of proteinuria following coronavirus (COVID)-19 vaccination

Table 3 summarizes the details of the treatment of exacerbation of proteinuria following COVID-19 vaccination. All 6 (100%) patients with new-onset NS and 15/21 (71.4%) patients with relapse were initiated on steroids or increased steroid dose. Figure 2 illustrates the duration between COVID-19 vaccination and the incidence of new-onset and relapse of NS; 15 cases (55.6%) were reported within 7 days of vaccination. Figure 3 illustrates the duration of new-onset and relapse of NS. In 7/21 (33.3%) patients with relapse of NS, proteinuria resolved within 7 days spontaneously. In all six (100%) patients with new-onset NS and 15/21 (71.4%) patients with relapse of NS, steroids were administered or their dose increased. Of them, proteinuria lasted for 7 days in seven (25.9%) patients and over 8 days in 20 (74.1%) patients. Tables 4 and 5 summarize the urinary abnormalities following COVID-19 vaccination. Nephrotic-range proteinuria was noted in 12/21 (57.1%) patients with no proteinuria before the vaccination. Hematuria was noted in 5/22 (22.7%) patients with no hematuria before the vaccination and was exacerbated in 3/5 (60.0%) patients with hematuria before the vaccination.

**Table 3:** Treatment after exacerbation of proteinuria after receiving COVID-19 vaccination

Treatments after this event (multiple answers allowed)	All cases (*n* = 27)	New-onset (*n* = 6)	Relapse (*n* = 21)
Started steroid	10	6	4
Increased steroid	11	0	11
Steroid pulse therapy	1	0	1
Started immunosuppressive therapy	3	1	2
Increased immunosuppressive therapy	4	2	2
Started RAS-I	1	0	1
No treatment	5	0	5

*COVID-19* coronavirus disease 2019, *RAS-I* renin–angiotensin system inhibitor

**Table 4:** Proteinuria after COVID-19 vaccination

Details of proteinuria	All cases (*n* = 27)	New-onset (*n* = 6)	Relapse (*n* = 21)
Cases with proteinuria before the vaccination of qualitative ( +) or higher or 0.3 g/day (g/g Cr) or higher	6	1	5
1.0 g/day (g/g Cr) ≤ proteinuria < 3.5 g/day (g/g Cr)	2	0	2
≥ 3.5 g/day (g/g Cr)	4	1	3
Cases with no proteinuria before the vaccination	21	5	16
1.0 g/day (g/g Cr) ≤ proteinuria < 3.5 g/day (g/g Cr)	8	0	8
≥ 3.5 g/day (g/g Cr)	13	5	8

*COVID-19* coronavirus disease 2019

**Table 5:** Hematuria after COVID-19 vaccination

Details of hematuria	All cases (*n* = 27)	New-onset (*n* = 6)	Relapse (*n* = 21)
Cases with hematuria before the vaccination	5	0	5
Exacerbated hematuria	3	0	3
Did not exacerbate hematuria	2	0	2
Cases with no hematuria before the vaccination	22	6	16
Appearance of hematuria	5	1	4
No hematuria	17	5	12

*COVID-19* coronavirus disease 2019

In the secondary survey, information regarding 5/5 patients was returned (response rate: 100%). Renal biopsy was performed after vaccination in one patient who was diagnosed with membranous nephropathy. Notably, five patients developed a slight and transient increase in serum creatinine levels, but none of the patients progressed to severe renal dysfunction.

## Discussion

We investigated the clinical characteristics of new-onset and relapse of NS following COVID-19 vaccination in Japan. Although 6 and 21 patients with new-onset and relapse of NS were reported, transiently increased serum levels of creatinine were noted in 5 patients. To the best of our knowledge, this is the first case series of new-onset and relapse of NS following COVID-19 vaccination in Japan.

To date, there have been several reports of new-onset and relapse of NS following COVID-19 vaccination [8–14, 20]. Vaccinations based on several mechanisms of triggering an immune response have been developed and administered; however, most cases of new-onset and relapse of NS have been reported with mRNA vaccination. Different COVID-19 vaccines utilize different methods to elicit host immunity. Pfizer-BioNTech [3] and Moderna vaccines [4] employ a lipid nanoparticle complexed with nucleoside-modified mRNA that encodes the SARS-CoV-2 spike protein (S protein), whereas the AstraZeneca vaccine employs an adenoviral vector that contains the gene sequence that encodes for the SARS-CoV-2 S protein [5]. These vaccines are designed to induce the host to synthesize the SARS-CoV-2 S protein, which in turn generates an effective immune response against the SARS-CoV-2 S protein. The induced T-cell-based response includes the upregulation of the production of cytokines, including interleukin-2, interferon-γ, and tumor necrosis factor-α, which can enhance B-cell production of immunoglobulins and trigger podocytopathies in predisposed patients [21–23]. These cytokines may play a role in exacerbating quiescent or subclinical glomerular diseases via a mechanism similar to that proposed for viral infections, which is a known trigger for de novo and relapsing glomerular diseases [24]. Actually, our survey showed new-onset and relapse of NS following COVID-19 vaccination in patients with not only MCD, but also various glomerular diseases, including focal segmental glomerulosclerosis, IgAN, membranous nephropathy, membranoproliferative glomerulonephritis, and C3 glomerulopathy, which is a rare kidney disorder [25]. Notably, in this study, one patient was newly diagnosed with membranous nephropathy following a kidney biopsy that was performed because of new-onset NS following COVID-19 vaccination, thus suggesting that such immune activation may be largely related to the mechanism of onset of glomerular diseases.

Acute kidney injury (AKI) is an important and consistent feature that has been reported in most cases of new-onset and relapse of NS following COVID-19 vaccination [8–11]. Although a causal association cannot be confirmed definitively, clinicians and pathologists should be aware that NS is a potential adverse effect of the vaccines. However, complete remission of NS and AKI can be achieved in most cases with the prompt initiation of steroid therapy. Post-vaccination kidney biopsy in patients with IgAN who developed gross hematuria revealed active endocapillary hypercellularity, leukocyte infiltration, fibrinoid necrosis, and crescents, although these lesions can involve only a minority of the glomeruli [17]. Gross hematuria typically resolves rapidly within days [17, 19]. These findings raise questions about the aggressiveness with which biopsy findings should be treated and whether flares are likely to be short-lived and transient following the vaccination. However, other cases of MCD with AKI have been reported to respond slowly to corticosteroids and require hospitalization in the intensive care unit to manage life-threatening fluid overload [8]. Currently, there are no guidelines regarding the second dose of the vaccine in such individuals. One potential option is to switch to a different COVID-19 vaccine to minimize the possibility of relapse. More experience in post-vaccination settings is needed to define the natural course and guide the optimal therapeutic management in the COVID-19 era.

Although the number of reported cases of vaccine-related NS is increasing, it represents a very small percentage of individuals who have been safely vaccinated. The risk of recurrence of glomerular diseases due to vaccination remains significantly lower than the risk of AKI requiring dialysis and/or death in individuals infected with COVID-19 [26]. Therefore, we recommend proceeding with COVID-19 vaccination in patients with glomerular diseases. Furthermore, it also proposes two important points of discussion for the patients. First, similar to the experience of patients who undergo transplantation, immunosuppressed patients with glomerular diseases may not mount a similar level of immune response and, consequently, achieve comparable levels of sustained immunity to the virus. Second, a small risk of recurrent disease exists up to a month after the second vaccination, during which the patient can self-monitor for danger signs and symptoms. Therefore, informed decision-making requires knowledge about the risk of infections, particularly against newer variants of COVID-19, in those who did not complete the vaccination schedule. Currently, the third dose vaccination has also begun in Japan. If yearly “booster” vaccines against COVID-19 are recommended, should patients who develop NS post-vaccination avoid repeat exposure to the vaccine? As previously mentioned, one potential option is to switch to a different COVID-19 vaccine to minimize the possibility of relapse since some patients have relapsed again on receiving vaccinations of the same type [8, 20, 24]. Although some of our case series had already been reported in detail as case reports [13, 14], it will require an understanding of the nature and severity of glomerular diseases in individual patients.

Our study has several limitations. First, there is a possibility of selection bias because the response rate was only 14.4% across 382 facilities. Furthermore, because the council members tend to be affiliated with large hospitals, our results did not include patients who were followed up in small clinics and rural hospitals. Second, because the questionnaire was a single survey, the clinical course in these patients could have varied depending on the timing of the response. Third, we were unable to collect detailed information on individual patients’ characteristics (such as age, CKD stage, and comorbidities) in the section of the survey that dealt with the treatment patterns and the patients’ outcomes. Therefore, a prospective cohort study in the future will be needed to overcome these limitations.

In conclusion, this small survey clarified the clinical features of new-onset and relapse of NS following COVID-19 vaccination in Japan. Although nephrologists should follow-up on the urinary findings carefully and periodically, it remains unclear whether COVID-19 mRNA vaccines are associated with the incidence of NS or whether NS coincides with mass vaccination. Additionally, it is not clear whether the second dose of the COVID-19 mRNA vaccine should be administered to patients who develop NS post-vaccination. Further studies are needed to determine the incidence of NS following COVID-19 vaccines and elucidate the pathophysiology of any incident glomerular injury.
